# Efficacy and Safety of Faricimab in Diabetic Macular Edema: Real-World Outcomes in Treatment-Naïve and Previously Treated Eyes

**DOI:** 10.3390/jcm14176173

**Published:** 2025-09-01

**Authors:** Olivia Esteban-Floría, Javier Mateo, Javier Lara, Isabel Bartolomé, Inmaculada Herrero, María A. Pérez, Concepción Cabello, Ana Honrubia, Isabel Pinilla, Javier Ascaso

**Affiliations:** 1 Department of Ophthalmology, Hospital Clínico Universitario Lozano Blesa, C. de San Juan Bosco, 15, 50009 Zaragoza, Spain; jmateo_1999@yahoo.com (J.M.); javierlara@me.com (J.L.); issbartolome@gmail.com (I.B.); herrero1118@gmail.com (I.H.); aperezinigo@hotmail.com (M.A.P.); conchacabello4@yahoo.es (C.C.); anahonrubia@telefonica.net (A.H.); ipinilla@unizar.es (I.P.); jascaso@gmail.com (J.A.); 2 Instituto de Investigación Sanitaria de Aragon (IIS), 50009 Zaragoza, Spain; 3 Faculty of Medicine, University of Zaragoza, 50009 Zaragoza, Spain

**Keywords:** diabetic macular edema, faricimab

## Abstract

**Background:** The objective of this study was to assess the efficacy and safety of faricimab in diabetic macular edema (DME) in patients who were treatment-naïve or previously treated in a real-world setting. **Methods:** This was a retrospective, observational, single-center study that included 105 eyes from 79 patients diagnosed with DME and treated with intravitreal faricimab between January 2024 and January 2025. Patients were categorized into two groups according to their treatment history, namely treatment-naïve eyes and eyes previously treated (switch group). Functional (best-corrected visual acuity, BCVA) and anatomical (central foveal thickness, CFT; macular volume, MV) outcomes were assessed. The safety of faricimab was evaluated from changes in intraocular pressure and the occurrence of adverse events. **Results:** BCVA improved significantly in both groups, with a mean gain of +0.16 in treatment-naïve eyes and +0.10 in switch eyes. The mean reduction in CFT was −53.7 µm in the naïve group and −37.8 µm in the switch group. MV decreased by −0.4 mm^3^ overall, with significant reductions in both groups. No adverse events were reported, confirming the safety of faricimab in routine clinical practice. **Conclusions:** Faricimab showed significant improvements in functional and anatomical outcomes in patients with DME, regardless of the use of previous anti-VEGF therapies. These findings support the effectiveness and safety of faricimab in a real-world clinical setting and reinforce its potential as a valuable treatment option for DME.

## 1. Introduction

Diabetic macular edema (DME) is a leading cause of vision loss in patients with diabetic retinopathy (DR) and is the most frequent manifestation of advanced-stage DR [1,2]. Approximately 5.5% of individuals with diabetes are affected by DME, making it an increasingly public health issue among working-age populations [3,4]. The pathophysiology of DME is a complex and multifactorial process that involves angiogenesis, increased retinal vascular permeability, inflammation and, ultimately, disruption of the blood–retinal barrier, driven by persistent hyperglycemia [2,4,5]. Vascular endothelial growth factor-A (VEGF-A) has been identified as a key mediator in this process, and anti-VEGF agents have become the first-line treatment for DME [5,6,7,8,9].

Several anti-VEGF agents, such as ranibizumab, aflibercept, and bevacizumab, have demonstrated efficacy in the treatment of DME in randomized clinical trials, including RISE and RIDE [8], VIVID and VISTA [10], and Protocol T [11]. These studies have shown significant improvements in both visual acuity and anatomical outcomes, supporting the use of anti-VEGF agents as a first-line therapy for DME [8,10,11]. However, the translation of these results into routine clinical practice faces several limitations. In real-world settings, incomplete response is common, driven by factors such as suboptimal adherence and the overall burden associated with frequent intravitreal injections and follow-up visits [6]. Additionally, a substantial proportion of patients experience incomplete or non-response to anti-VEGF monotherapy [12,13]. Persistent DME and/or limited visual improvement have been reported in approximately 20% of patients, who are considered refractory to anti-VEGF-A agents [13]. This underscores the need for alternative treatment strategies.

Faricimab (Vabysmo^®^, Roche Pharma AG, Grenzach-Wyhlen, Germany) is a novel therapeutic option consisting of a bispecific antibody targeting both VEGF-A and angiopoietin-2 (Ang-2) [5]. Ang-2 has also been identified as a key mediator of DME pathogenesis, promoting endothelial and vascular destabilization and leakage through inhibition of the Tie2 signaling pathway. This signaling pathway is specific to vascular cells as is the VEGF pathway and more active in promoting endothelial cell survival, maturation, and stability. Acting together with the VEGF signaling pathway, it represents a novel therapeutic target for diabetic macular edema [5,12].

This dual mechanism may enhance anatomical and functional outcomes while reducing treatment burden. The YOSEMITE and RHINE phase III randomized clinical trials were designed to evaluate patient outcomes and treatment burden in patients with DME treated with faricimab. The results demonstrated significant improvements in visual acuity and anatomical outcomes while allowing for extended dosing intervals of up to 16 weeks (Pollreisz A et al. IOVS 2023;64:ARVO Annual Meeting Abstract 2817) [14]. However, outcomes obtained in real-world settings often differ from those observed in clinical trials, which include highly selected patient populations, strict inclusion criteria, and tightly controlled conditions [15]. In this context, there is a need to assess the performance of faricimab in broader, more heterogeneous patient populations managed in clinical practice.

The purpose of this study is to provide real-world evidence on the efficacy and safety of faricimab in a tertiary care setting, comparing functional and anatomical outcomes in treatment-naïve versus previously treated eyes.

## 2. Materials and Methods

### 2.1. Study Design

For this retrospective, observational, single-center study, medical records of consecutive patients with a diagnosis of DME who received intravitreal faricimab (IVF) at the Department of Ophthalmology of University Hospital Lozano Blesa between 1 January 2024 and 31 January 2025 were reviewed. The research protocol adhered to the Declaration of Helsinki, and the study received approval (C.I. EOM25/027) from the local ethics committee (Comité Ético de Investigación Clínica de Aragón [CEICA]).

### 2.2. Inclusion and Exclusion Criteria

Participants were adults (≥18 years) with a confirmed diagnosis of type II diabetes mellitus and DME in center subfield retinal thickness, including focal or diffuse macular thickening, cystoid macular edema (fluid-filled spaces within the retina), serous retinal detachment, and/or exudative changes with intraretinal and/or subretinal fluid in the macular area, confirmed by spectral-domain optical coherence tomography (SD-OCT). Only patients who had received at least one IVF injection and had undergone a minimum follow-up period of 4 months after treatment initiation were included. The exclusion criteria were no diabetic macular edema despite other diabetic findings in the macular area (hyperreflective dots or disruption of the ellipsoid zone), vitreoretinal interface disorders in the epiretinal membrane or vitreomacular traction and other retinal comorbidities, such as retinal vein occlusion or age-related macular degeneration, a history of previous vitrectomy, and severe media opacities. Patients with incomplete medical records that precluded reliable data extraction or incomplete follow-up were also excluded from the study.

### 2.3. Treatment Protocol

Included eyes were stratified into 2 groups based on previous treatment history. The treatment-naïve group comprised eyes that received faricimab as first-line anti-VEGF therapy. The switch group included eyes previously treated with aflibercept, ranibizumab, and/or corticosteroid, in which the decision to initiate faricimab was based on suboptimal anatomical response to previous therapy (less than 25% reduction in central retinal thickness (CRT) after the loading phase, remaining above 300 µm or showing persistence or increase in retinal fluid after 4 weeks of previous injection).

All patients followed a standardized treatment protocol consisting of 3 monthly loading doses of IVF (6.0 mg/0.05 mL) (loading phase). Following this initial phase, a treat-and-extend (TAE) regimen was applied. Injection intervals were adjusted in 4-week increments based on anatomical response and functional improvement. The maximum extension allowed was 16 weeks. All injections were administered under sterile conditions in a dedicated procedure room by experienced retina specialists.

### 2.4. Data Collection

The following data were collected for the analysis: demographic variables (age, sex), prior anti-VEGF and corticosteroid injections, baseline best-corrected visual acuity (BCVA), baseline central foveal thickness (CFT), and baseline macular volume (MV). The primary outcome was changes in decimal BCVA. Secondary outcomes were changes in CFT and MV, determined by SPECTRALIS OCT (Heidelberg Engineering, Heidelberg, Germany).

A safety event was considered an ocular complication as sustained intraocular pressure elevation, intraocular inflammation, endophthalmitis, retinal detachment or vitreous hemorrhage, or a systemic adverse event as a cardiovascular or cerebrovascular event. Safety events were monitored at each visit through intraocular pressure measurement, biomicroscopy, and review of recent systemic medical history.

### 2.5. Statistical Analysis

Data analysis was performed using SPSS version 22.0 (IBM Corp, Chicago, IL, USA) and Stata version 15.1 (StataCorp LLC, College Station, TX, USA). Quantitative variables were reported as mean ± standard deviation (SD). Paired *t*-tests were used to compare changes from baseline to post-treatment. To estimate the effect size of these changes, Cohen’s *d* was calculated, with thresholds interpreted as small (0.2), medium (0.5), and large (0.8). Test results were considered statistically significant for *p*-values < 0.05.

## 3. Results

### 3.1. Baseline Characteristics

A total of 123 eyes from 94 patients diagnosed with DME were initially screened for eligibility. Eighteen eyes (14.6%) from fifteen patients were excluded during the study due to the development of different pathologies, including severe cataract (*n* = 4), prior central vein occlusion (*n* = 1), history of glaucoma (*n* = 6), incomplete clinical data (*n* = 2), or loss to follow-up (*n* = 5), resulting in 105 eyes from 79 patients being included in the analysis.

Patients were stratified into two groups based on previous treatment: 28 eyes (26.7%) were included in the treatment-naïve group and 77 eyes (73.3%) in the switch group that had previously received aflibercept or ranibizumab. In total, 22 out of 77 eyes in the switch group had previously received both anti-VEGF and corticoisteroid injections.

Baseline demographic, functional, and anatomical characteristics are summarized in Table 1. The mean age was 68.0 ± 8.6 years, with a higher proportion of males (74%), and the mean follow-up time was 10.0 ± 3.1 months. Baseline functional and anatomical parameters did not differ significantly between treatment-naïve and switch groups. All eyes in both groups presented with non-proliferative DR at baseline. Eleven eyes from the switch group had a history of panretinal photocoagulation prior to the initiation of faricimab therapy.

### 3.2. Visual Outcomes

BCVA improved significantly after IVF (Table 2). For the entire cohort, the mean gain in BCVA was +0.07, from 0.49 ± 0.28 at baseline to 0.56 ± 0.28 at the final visit (*p* < 0.001; Cohen’s *d* = 0.49). Subgroup analysis showed a greater improvement in treatment-naïve eyes, with a significant increase in BCVA from 0.59 ± 0.11 to 0.75 ± 0.29 (mean gain +0.16; *p* < 0.003; Cohen’s *d* = 0.53) compared to the switch group, which showed a significant increase from 0.46 ± 0.21 to 0.56 ± 0.24 (mean gain +0.10, *p* = 0.026; Cohen’s *d* = 0.43).

### 3.3. Anatomical Outcomes

Anatomical outcomes improved consistently across the cohort (Table 3). The overall mean CFT decreased by −45.7 μm (*p* < 0.001; Cohen’s *d* = 0.83), from 338.9 ± 87.5 µm at baseline to 293.2 ± 62.4 µm at the final visit. A greater effect was observed in treatment-naïve eyes (−53.7 µm, from 350.1 ± 87.9 to 296.4 ± 64.3 µm; *p* < 0.001; Cohen’s *d* = 1.08) compared to switch eyes (−37.8 µm, from 328.0 ± 87.2 to 290.2 ± 61.3 µm; *p* = 0.001; Cohen’s *d*= 0.63). All naïve eyes and almost 70% of switch eyes had no diabetic macular edema in CFT in the last OCT during follow-up.

MV was also significantly reduced after faricimab therapy (Table 4). In the total cohort, MV decreased from 9.1 ± 0.8 mm^3^ at baseline to 8.7 ± 0.7 mm^3^ at the final visit (mean change −0.4 mm^3^; *p* < 0.001; Cohen’s *d* = 0.97). Both subgroups experienced statistically significant reductions: naïve eyes showed a mean decrease of −0.4 mm^3^ (from 9.3 ± 0.9 to 8.8 ± 0.9 mm^3^; *p* < 0.001; Cohen’s *d* = 0.84), while switch eyes showed a reduction of −0.5 mm^3^ (from 9.1 ± 0.7 to 8.6 ± 0.6 mm^3^; *p* < 0.001; Cohen’s *d* = 1.11). In this case, none of the naïve eyes and almost 55% of switch eyes presented macular thickening in the last examination.

### 3.4. Treatment Exposure and Safety

The number of IVF injections per eye during the study period was 5.7 ± 2.8. Of the 105 included eyes, 47.6% (*n* = 50) were followed up until the loading phase and 52.4% (*n* = 55) continued treatment under a TAE regimen. Following the loading phase, all treatment-naïve eyes showed a reduction in CFT greater than 20%, and complete resolution of macular edema was observed in all switch eyes. All eyes were eligible for extension to a 4-week interval following the loading phase. At the final visit, 30 eyes (54.5%) reached a dosing interval of 8 weeks, while 25 eyes (45.5%) were extended to intervals between 12 and 16 weeks.

During the follow-up period, no adverse events were reported in any patient, consistent with the safety profile of faricimab, as established both in clinical trials and recent real-world studies [12,13,14,16,17,18,19,20,21].

## 4. Discussion

This retrospective study evaluated the efficacy and safety of IVF in a real-world setting in patients with DME, whether treatment-naïve or previously treated, and improvements were observed in both functional and anatomical outcomes while a favorable safety profile was maintained. These findings are similar to other studies in real-word practice [22], reinforcing evidence from pivotal trials in a more heterogeneous and clinically representative population.

Before initiating faricimab treatment, our patients were treated with anti-VEGF and/or corticosteroid in a pro re nata regimen. Inclusion in a TAE protocol allowed for a proactive and personalized dosing strategy, based on disease activity in each individual case. Our TAE protocol enabled tailored injection intervals, with 52.4% of eyes reaching intervals of 8 weeks or longer. This aligns with Ohara et al. (2023), who reported ≥12-week intervals in 44.4% of their cohort [17]. This approach ensures effective anatomical control while minimizing the treatment burden by extending injection intervals when clinically recommended.

In our cohort, we observed significant improvements in both functional and anatomical outcomes after faricimab treatment. CFT was significantly reduced by 45.7 µm in the overall population, with a more pronounced decrease in treatment-naïve eyes compared to previously treated ones. This anatomical response highlights the efficacy of dual VEGF-A and Ang-2 blockade for the control of DME, as reported in previous real-world studies, which also described significant reductions in CFT after faricimab treatment [13,16,21]. At the functional level, the mean visual acuity gain was +0.07 in the total cohort, with greater improvement in the naïve group (+0.16) compared to the switch group (+0.10), consistent with findings from other real-world studies [23]. Although a better functional response in treatment-naïve eyes was expected, the gain observed in previously treated eyes supports the potential of IVF in providing visual benefits even in advanced or refractory cases. These findings are particularly relevant considering that in real-world settings, patients often receive fewer injections and follow more flexible anti-VEGF regimens than in clinical trials, which may contribute to suboptimal responses [24].

Although the shorter follow-up period in the naïve group should be considered when interpreting long-term outcomes, particularly regarding treatment interval extension and recurrence monitoring, follow-up periods in both groups are longer than in other real-word studies [22]. Therefore, the inclusion and the results of both treatment-naïve and switch groups provide added value to our analysis and reinforce the utility of faricimab across diverse patient profiles.

We also analyzed MV, which offers a more comprehensive view of the macula status compared to foveal retinal measurements. Foveal retinal thickness, although useful, may not fully reflect the extent of macular edema, as it does not account for the entire macula region outside the central fovea. Moreover, it can be influenced by factors, such as age, sex, and refractive status, and it may be confounded by retinal atrophy secondary to DR. These limitations can affect its diagnostic sensitivity [25]. MV, in contrast, provides a representative measure of the total fluid volume within the macular region, offering a more robust estimation of edema severity. In this context, You et al. (2021) found that central macular fluid volume (CMFV), quantified by OCT angiography, had greater diagnostic accuracy for DME than central subfield thickness [25]. In our cohort, we observed a significant reduction in MV in both groups (*p* < 0.001), reinforcing the anatomical efficacy of faricimab. The inclusion of MV in the analysis adds value to the study by complementing CFT assessment and supporting its potential utility as a biomarker for the evaluation of DME severity and treatment response in future research.

As Huber et al. in 2024 and Talks et al. in 2019 pointed out, trial populations differ from real-world cohorts, where adherence, comorbidities, and treatment patterns vary widely [12,15]. Our outcomes are in line with those of the YOSEMITE and RHINE phase III trials but reflect a more diverse clinical context that includes patients with variable treatment patterns. And our results are also in line with recent real-world evidence on faricimab, which has shown consistent functional and anatomical improvements and allowed for extended treatment intervals in both treatment-naïve and previously treated DME eyes [12,16,26,27]. Unlike many previous reports, our study implemented a structured TAE protocol, which reflects the most commonly used treatment regimen in routine clinical practice. As previously discussed, we also evaluated MV as an anatomical outcome, which is rarely reported in observational studies and further distinguishes the scope of our analysis.

Generating evidence from routine clinical practice is essential to better understand the behavior of faricimab beyond the controlled trial setting, particularly in previously treated and clinically complex patients. In this context, our study expands the current understanding of the utility of this drug in heterogeneous populations.

Our work had some limitations that are inherent to observational studies. First, the retrospective analysis could affect consistency on data collection and limit control of confounding variables. Second, there was no control group to compare the progress of patients with or without intervention. Moreover, the single-center nature of the study warrants caution when extrapolating the results to different clinical contexts. Unlike other real-world studies, which stratified patients based on baseline response [19], this study classified them by prior anti-VEGF therapy, which could impact the interpretation of visual outcomes due to clinical heterogeneity within the groups. Although the results should be interpreted with caution, the observations obtained remain highly relevant for clinical practice. This study comprises one of the largest cohorts published to date on faricimab in real-world settings, validating the applicability of its findings.

## 5. Conclusions

This real-world study confirms the use of IVF as an effective and safe treatment option for patients with DME, regardless of prior therapy status. Together, these findings illustrate the dual benefit of faricimab in improving functional and anatomical outcomes while reducing treatment burden through a TAE regimen, and they highlight the added value of MV assessment. The absence of observed adverse events supports the favorable safety profile of faricimab in routine clinical practice, consistent with findings from clinical trials and other real-world studies [12,13,16,17,18,19,20,21]. These data further show the good tolerability of faricimab even in unselected, real-world populations. Overall, the results support the integration of IVF into the current DME therapeutic landscape and underline the need for further research in real-world settings.

## Figures and Tables

**Table 1:** Baseline demographic and clinical characteristics.

Parameter *	Treatment-Naïve (*n* = 28)	Switch (*n* = 77)	Total (*n* = 105)
Age (years)	66.4 ± 9.1	68.7 ± 8.4	68.0 ± 8.6
Male/female (%)	71/29	75/25	74/26
Mean prior anti-VEGF injection number (*n*)	0	12.2 ± 5.9	
Mean prior corticosteroid injection number (*n*)	0	3.5 ± 0.6	
Baseline BCVA (decimal)	0.59 ± 0.11	0.46 ± 0.21	0.49 ± 0.28
Baseline CFT (μm)	350.1 ± 87.9	328.0 ± 87.2	338.9 ± 87.5
Baseline MV (mm^3^)	9.3 ± 0.9	9.1 ± 0.7	9.1 ± 0.8
Mean follow-up period (months)	8 ± 1.3	12 ± 4.5	10 ± 3.1

* Continuous variables are presented as mean ± SD. Categorical variables are reported as counts. BCVA: best-corrected visual acuity; CFT: central foveal thickness; MV: macular volume.

**Table 2:** Change in best-corrected visual acuity (decimal) after faricimab treatment.

Group	Baseline BCVA	Final BCVA	Mean Change	*p*-Value	Cohen’s *d*
Treatment-naïve	0.59 ± 0.11	0.75 ± 0.29	+0.16	0.003	0.53
Switch	0.46 ± 0.21	0.56 ± 0.24	+0.10	0.026	0.43
Overall	0.49 ± 0.28	0.56 ± 0.28	+0.07	<0.001	0.49

Results are presented as mean ± SD. BCVA: best-corrected visual acuity.

**Table 3:** Change in central foveal thickness after faricimab treatment.

Group	Baseline CFT (μm)	Final CFT (μm)	Mean Change	*p*-Value	Cohen’s *d*
Treatment-naïve	350.1 ± 87.9	296.4 ± 64.3	−53.7	<0.001	1.08
Switch	328.0 ± 87.2	290.2 ± 61.3	−37.8	0.001	0.63
Overall	338.9 ± 87.5	293.2 ± 62.4	−45.7	<0.001	0.83

Results are presented as mean ± SD. CFT: central foveal thickness.

**Table 4:** Change in macular volume after faricimab treatment.

Group	Baseline MV (mm^3^)	Final MV (mm^3^)	Mean Change	*p*-Value	Cohen’s *d*
Treatment-naïve	9.3 ± 0.9	8.8 ± 0.9	−0.4	<0.001	0.84
Switch	9.1 ± 0.7	8.6 ± 0.6	−0.5	<0.001	1.11
Overall	9.1 ± 0.8	8.7 ± 0.7	−0.4	<0.001	0.97

Results are presented as mean ± SD. MV: macular volume.

## Data Availability

Only if someone need the data, I can send via email.
